# Minding the gaps: health financing, universal health coverage and gender

**DOI:** 10.1093/heapol/czx063

**Published:** 2017-07-25

**Authors:** Sophie Witter, Veloshnee Govender, TK Sundari Ravindran, Robert Yates

**Affiliations:** 1 Institute for Global Health and Development, Queen Margaret University, Edinburgh, UK; 2 Health Economics Unit, School of Public Health and Family Medicine, University of Cape Town, Cape Town, South Africa; 3 Sree Chitra Tirunal Institute for Medical Sciences and Technology, Kerala, India; 4 Centre on Global Health Security Chatham House, The Royal Institute of International Affairs, 10 St James's Square, London, SW1Y 4LE UK

**Keywords:** health financing, gender, universal health coverage, India

## Abstract

In a webinar in 2015 on health financing and gender, the question was raised why we need to focus on gender, given that a well-functioning system moving towards Universal Health Coverage (UHC) will automatically be equitable and gender balanced. This article provides a reflection on this question from a panel of health financing and gender experts.

We trace the evidence of how health-financing reforms have impacted gender and health access through a general literature review and a more detailed case-study of India. We find that unless explicit attention is paid to gender and its intersectionality with other social stratifications, through explicit protection and careful linking of benefits to needs of target populations (e.g. poor women, unemployed men, female-headed households), movement towards UHC can fail to achieve gender balance or improve equity, and may even exacerbate gender inequity. Political trade-offs are made on the road to UHC and the needs of less powerful groups, which can include women and children, are not necessarily given priority.

We identify the need for closer collaboration between health economists and gender experts, and highlight a number of research gaps in this field which should be addressed. While some aspects of cost sharing and some analysis of expenditure on maternal and child health have been analysed from a gender perspective, there is a much richer set of research questions to be explored to guide policy making. Given the political nature of UHC decisions, political economy as well as technical research should be prioritized.

We conclude that countries should adopt an equitable approach towards achieving UHC and, therefore, prioritize high-need groups and those requiring additional financial protection, in particular women and children. This constitutes the ‘progressive universalism’ advocated for by the 2013 Lancet Commission on Investing in Health.

Key Messages

- Insufficient attention has been paid to the interaction of gender and health financing; we call for better collaboration to fill this gap.
- While Universal Health Coverage (UHC) emphasizes equity, some groups have higher health needs and lower financing capabilities than others; this implies the need for progressive universalism, which puts the needs of vulnerable groups like women and children first.
- Broad recommendations from our review include public financing of health care services with resources mobilized from progressive taxation of income and wealth; firm action by governments to regulate the private health sector, especially in the area of price controls; attention to coverage of different groups of women when implementing health financing reforms; and social protection schemes that go beyond women from households below the poverty line and with packages covering more than maternal health.
- The underlying political and social determinants that undermine access for vulnerable and marginalized groups (e.g. poor indigenous women, adolescents) must also be tackled to achieve the broader equity and effectiveness goals of UHC.

## Introduction

The world is rallying round Universal Health Coverage (UHC) as a common health goal, whereby everybody receives the quality health services they need without suffering financial hardship (WHO 2010a). UHC has been incorporated as a sub-goal within the overall health Sustainable Development Goals (SDG) agreed at the special UN development summit in September 2015 (United Nations 2015). Many of the leading health agencies (including WHO and the World Bank) are promoting UHC as the best strategy to achieve the overall health SDG.

UHC is built on the notion of equity (United Nations 2015). First, the universal aspect of the concept clearly indicates that everybody should be covered—nobody should be left behind. Second, it states that health services should be allocated according to people’s needs, which implies that people with higher needs (e.g. pregnant women, young children and the chronically sick) should receive more services than others. Finally, the financial protection component of UHC implies that people’s financial contributions towards funding health services should be according to their ability to pay. UHC, therefore, requires that healthy and wealthy members of society cross-subsidise services for the sick, the vulnerable and the poor.

One only has to look at health indicators across the world to see that we are a long way from achieving this goal. The World Bank and WHO have estimated that around 400 million people lack access to basic health services, and that 6% of people in low- and middle-income countries are tipped into or pushed further into extreme poverty because of health spending (World health Organization 2015). Also, it is clear in many countries large inequalities in health outcomes and service coverage rates persist between different population groups, indicating that the equity principles entrenched in UHC are not being fulfilled (Ruger and Kim 2006; Oxfam 2013). In disaggregating health coverage data, one group which is often shown to be disadvantaged are women, who through their life-cycle often have greater healthcare needs than men but who, due to economic inequalities, often have a lower ability to pay for services (World Health Organization 2016). According to the definition of UHC, many women ought to be the beneficiaries of cross-subsidies from more privileged groups in society in accessing health services, but this is clearly not happening at sufficient scale (Nanda 2002; Oxfam 2013).

Achieving genuine universal health coverage ought to help reduce these inequalities, and will, therefore, not only represent a means to achieve the health SDG but also to make progress towards SDG 5 on achieving gender equity. It is, therefore, to be welcomed that the world is now committed to UHC; however, how this is achieved is vitally important as some routes are likely to be more equitable than others in closing gender gaps in coverage. As countries plan and implement strategies to reach UHC they will need to address a broad range of systems reforms, involving all of the health systems ‘building blocks’ identified by WHO (WHO 2007) including governance, health care financing, health workforce, medical products and technologies, information and research, and service delivery. However, as WHO identified itself in its World Health Report in 2010, the area that is likely to have the greatest impact on improving equity will concern reforming the health financing system (WHO 2010a).

While the financing of healthcare clearly influences its demand, access and utilization are also influenced by a diverse range of other factors operating on the patient’s side (i.e. demand-side). Social stratifications and inequalities (e.g. socio-economic status, gender, ethnicity, race, caste) and their intersections with each other (e.g. African-American men in the United States, scheduled-caste women in India) are additional influences which might shape men’s and women’s access to household and societal resources and their perceptions and experiences of the cultural acceptability of services (Larson et al. 2016a,b). There is a substantial body of literature across a range of different contextual settings indicating that men and women are different in the ways they perceive and experience illness, and ultimately seek care for a range of curative and promotive health services (Gao and Yao 2006; Batnitzky 2008; Braitstein *et al.* 2008; Mwachofi 2009; Ringheim 2011). There is less literature, however, exploring the gendered effects of different financing mechanisms, and how these affect healthcare access and utilization (Ravindran and de Pinho 2005; Sen and Govender 2015).

The purpose of this paper is to highlight the gaps in the health systems literature in relation to health financing and gender, and to explore which financing reforms are likely to be the most effective at accelerating progress toward UHC while at the same time addressing gender inequities (Research in Gender and Ethics 2015). While evidence is drawn from the overall literature, we utilize a case study of India in order to showcase a country where health-financing reforms are having an impact on gender inequities. The paper concludes by calling for greater collaboration between gender and health financing experts.

## Methods

This paper draws on the expertise of the authors and on a rapid review of the health literature (grey and peer-reviewed articles). Although there are bodies of work on related topics in the economics and wider development literature, our interest was to capture the extent to which health practitioners had researched and reflected on this topic, so our focus was on health literature. With respect to universal health coverage, there were several primary documents and reports (i.e. grey literature) that were drawn on. These included WHO reports (i.e. 2010 report on universal coverage, the 2008 report on primary health care) and technical reports (Carrin and James 2004). With respect to gender and women, primary documents included reports by the United Nations ([Bibr czx063-B53][Bibr czx063-B54]). In addition, we included reports and publications focusing on the gender implications of health financing (Witter and Ensor 2012), health-financing reforms (WHO 2010 b) and universal coverage (Witter and Ensor 2012; Sen and Govender 2015) that were relevant to this paper. The reference lists of these documents were also reviewed and relevant articles identified for inclusion in this paper.

For published articles, both empirical and review, searches were conducted on PubMED and Google Scholar as well as specific journals focusing on gender and sexual and reproductive health (e.g. Reproductive Health Matters) and health policy (e.g. Health Policy and Planning, WHO Bulletin). The following key words were used for searches: ‘universal health coverage’, ‘universal coverage’, ‘health care financing’, ‘health financing reforms’, ‘insurance’, ‘community-based insurance’, ‘demand-side financing’, ‘gender’, ‘women’, ‘maternal health’, ‘sexual and reproductive health’, ‘access’, ‘equity’, ‘equality’ and ‘efficiency’. The search limits included all articles published since 2000 in English.

The India case study was based on published articles and reports about health financing in India since 2000 available from the same databases and journals, and key Indian sources publishing policy related articles (e.g. Economic and Political Weekly). The key words for the search included, in addition to those already mentioned, names of specific health financing schemes (e.g. Rashtriya Swasthya Bhima Yojana (RSBY)).

## Results

In its seminal World Health Report (WHO 2010a), WHO looks at three main functions of a health financing system: how revenues for health services are collected, how they are pooled to spread financial risks and what purchasing mechanisms are used to pay for health services. This article looks at these functions in turn to assess the extent of research into gender equity considerations in each area. The findings are presented below, grouping the first two functions (revenue collection and pooling) which are heavily interrelated.

## Overview of evidence on gendered effects of revenue collection and pooling for health care

This section explores the evidence on gendered effects of the health financing functions identified by WHO as revenue collection and pooling and how these affect healthcare access and utilization.

One can classify the main health-financing mechanisms into two broad groups—those that are private and voluntary in nature and those that are public and compulsory (WHO 2010a). With the first group, individuals and households have a large degree of choice as to whether and how much they choose to contribute towards the health financing system. The main mechanisms here include direct patient fees, voluntary private insurance schemes, voluntary health savings accounts and personal philanthropic aid.

In the latter group, there is a tendency for the state to compel people to make contributions and specify how much they pay, with the rich often having to contribute more than the poor. The main mechanisms here include financing from general taxation, compulsory social health insurance, mandatory health savings accounts and overseas development assistance. These groupings of financing mechanisms have different capacities to pool resources and in particular perform very differently against an equity measure of being able to facilitate cross-subsidies to poor and vulnerable groups in society.

### Private, voluntary mechanisms

#### User fees

User fees are out-of-pocket (OOP) payments which users pay for services at the point of use. OOP payments have been described by WHO (2008, p24) as ‘…the most inequitable method for financing healthcare services’. There are clear gender implications related to user fees, which have been shown to affect men and women differently. In many contexts, across both high- and low-income countries, for example, gender biases operating at the societal and household level often mean that women have less voice in the control and distribution of how household resources are shared among household members (United Nations 2009). In such contexts, user fees limit women's access to health care due to their lack of control over financial resources, the implications of which are expanded as a result of their greater reproductive healthcare needs (WHO 2010 b). According to WHO (2010a, p. 23), for example, [w]omen incur more out-of-pocket payments than men…paying for delivery care and other reproductive health services places a higher financial burden on women…[and] out-of-pocket expenditure may prevent more women than men from utilizing essential services.

Additionally, gender intersects with other social stratifications (e.g. socio-economic status, race, ethnicity, caste, age), further challenging access to and utilization of care (Ravindran 2012). Studies in Africa and Asia, for example, indicate that user fees severely constrain access to healthcare for the most vulnerable (such as elderly men and women, widows and women who are heads of their households) (Balagopal 2009; Onah and Govender 2014). In contexts where women and their families are required to pay for reproductive health services, delivery and obstetric care has been found to be unaffordable, even catastrophic (Parkhurst *et al.* 2005; Honda *et al.* 2011). A study covering Mombasa in Kenya and Mysore in India (2012–13) confirms the highly regressive nature of spending on sexual and reproductive health services. The poorest households spent 2 times as much and 10 times as much as the least poor in India and Kenya, respectively (Haghparast-Bidgoli *et al.* 2015).

When user fees have either been completely abolished or removed for selected services, utilization and access have improved, and in some instances key health indicators have improved. For example, significant improvements were recorded in utilization of maternity services and maternal mortality rates declined when user fees were removed for pregnant women in Ghana (Donnelly 2015).

The removal of user fees on its own, however, does not inevitably lead to improvements in healthcare access, and quality of care issues often persist (Schneider and Gilson 1999; Parkhurst *et al.* 2005; De Allegri *et al.* 2011). In South Africa, free maternal health care and the introduction of the Termination of Pregnancy bill within the public sector were important policies for improving healthcare access and improving maternal health, however, acceptability and quality of care remained a challenge (Schneider and Gilson 1999). A recent four-country study of obstetric fee exemption policies found that there are high risks of favouring better off households unless exemption policies are accompanied by concerted efforts to address other barriers, such as physical and cultural and those related to perceptions of quality of care. It also emphasized the need to address underlying systemic weaknesses, including in stewardship, and to embed exemption policies in an overall national plan to achieve UHC (Witter *et al.* 2016).

#### Private and voluntary health insurance

Evidence from both high- (e.g. USA) and middle-income countries (e.g. South Africa) with significant private health insurance coverage indicates that private health insurance is inequitable by excluding the unemployed and socio-economically disadvantaged (Govender *et al.* 2014). A study of privately insured households in South Africa found that almost half of privately insured households were partially insured,^1^ and ‘on average, more household heads in partially insured households were female, unmarried, with primary school education or no education, Black and unemployed’ (Govender *et al.* 2014). In the Indian context, ‘household members within male-headed households were twice as likely to be insured as those in female-headed households,’ with implications for healthcare access. Voluntary, community-based health insurance schemes which intend to meet the gap in insurance coverage in the informal sector through low premia, targeting women, the poor and rural populations, have also been unable to provide coverage for those ‘without access to cash–including the elderly and women from non-poor households’ (Ravindran 2012).

The need for explicit legislative measures to prevent gender rating in private health insurance is brought home by the experience of the USA. Gender rating is the practice of charging different rates for identical health services on the basis of gender. There is little known about how widespread gender rating is in LMICs. Before the Affordable Care Act was implemented in the USA, gender rating in individual plans caused women to pay an estimated US$1 billion more annually than men. Further, only 12% of individual plans included maternity benefits, and many preventive sexual and reproductive health services were not covered. The Affordable Care Act implemented since 2014 has made gender-rating illegal and also included a range of sexual and reproductive health services including contraception, screening and counselling for domestic and interpersonal violence, mammograms and colonoscopy. These benefits are now under threat from plans to reform Obamacare.

### Compulsory public mechanisms

Countries such as Thailand (Tangcharoensathien *et al.* 2014) and Mexico (Ibáñez and Garita 2015) have made rapid progress towards universal coverage through a combination of social health insurance, which covers those who are formally employed and salaried, and tax revenue, which covers the financial contributions of those who are economically vulnerable (i.e. poor, children, elderly, informal sector). The evidence from both these countries indicates that universal coverage has been effective in reducing financial costs of health care for the economically vulnerable; however, important challenges persist in both these contexts, particularly in relation to sexual and reproductive health (SRH) services.

In Mexico, inadequate resourcing of SRH services (i.e. distribution of services and health personnel including midwives), alongside ‘inequalities affecting women's access to health services, especially those that are based on income, age, ethnic origin and geographical residence’ (Ibáñez and Garita 2015, 244), have contributed to unsatisfactory progress in reducing maternal mortality and adolescent pregnancy rates. In comparison, Thailand’s maternal health picture is considerably better since explicit attention and effort was made in including ‘almost all relevant SRH services envisioned in the Programme of Action (POA) of the International Conference on Population and Development (ICPD), including treatment of reproductive tract cancers in the UHC benefit package’ (Tangcharoensathien *et al.* 2014, p. 246). Despite this progress, adolescent pregnancy rates have increased, access to safe abortions remains a challenge, and gender-based violence continues to be a major societal and public health challenge. Key messages emerging from both of these countries are that progress towards UHC in terms of developing effective financing mechanisms needs to be accompanied by (1) attention to services which predominately affect women, such as SRH, and (2) efforts to tackle the underlying political and social determinants that undermine access for vulnerable and marginalized groups (such as poor indigenous women and adolescents).

In many countries, health insurance is mandatory in theory but remains voluntary in practice for the informal sector. In these countries, many of the problems of voluntary financing schemes recur. Dixon (2014), for example, illustrates the intensely gendered nature of health insurance enrolment through her study of the National Health Insurance Scheme in Ghana. Major factors determining enrolment and dropping out were wealth, education and desire for health insurance. However, while only the poorest men were more likely to never enrol, wealth was a determinant of enrolment for women across the wealth spectrum, with the poorest women >6 times less likely to enrol as the wealthiest and even women from the middle wealth group almost 2.5 times less likely to enrol. Women with children under five and living in non-nuclear households were more likely to drop-out, and the three-month *block-out* time before reactivating coverage for premium defaulters was likely to penalize women for their mothering and family-related responsibilities (Dixon 2014).

### Development assistance

Development assistance accounts for an average of 25% of overall financing for health care in low-income countries (WHO 2015a). In the health sector, the OECD estimates that 51% of total bilateral aid to health focuses on gender equity, largely through investments in basic health care, such as primary health care programmes and health education. Support for family planning and reproductive health care made up a very small share of total gender equity focussed aid in the health sector OECD 2013 despite its potential contribution to MDG 5, focused on reducing maternal deaths, which was one of the least performing MDG goals (WHO 2015a).

Aid effectiveness studies have found a link between overall volumes of aid and improved outcomes, including reduced maternal mortality; however, improved outcomes are strongly affected by domestic conditions, including increases in the volume of domestic financing allocated for health and education (RECOM 2016).

## Overview of evidence on gendered effects of health purchasing

### Resource allocation

There have been some attempts to track allocation of resources to RMNCH through sub-accounts within national health accounts, as a part of the tracking of international commitments made to women and children’s health (WHO 2012). However, wider gendered analyses of health financing resource allocation are limited.

### Purchasing and benefits packages

In recent years, there have been a number of initiatives to channel publicly sourced financing resources (usually from taxation and aid financing) to target populations (e.g. low socio-economic, pregnant women and children) to increase their capacity to purchase RMNCH health services. This demand-side financing, as opposed to supply-side financing of services, has often used mechanisms such as vouchers or conditional cash transfers (Handa and Davis 2006; Lim *et al.* 2010; Ahmed and Khan 2011).

The overall experience from both South America and Asia has been that demand-side financing, which has primarily focused on maternal and child health, can be effective in reducing the financial barriers to access, increasing utilization of prioritized health services (Witter and Somanathan 2012). However, there have been concerns from a range of settings about the need for adopting supply-side interventions to improve the performance of demand-side interventions. For example, a demand-side strategy may not be very effective without significant expansion of the service delivery capacity of health facilities at the sub-district level (Handa & Davis 2006; Barber 2010; Ahmed and Khan 2011). Moreover, work in Uganda has shown that while demand-side financing, such as vouchers, has improved access to maternal health services, it does not address the underlying causes (such as negative gender power relations) affecting women’s ability to pay for and access services (Morgan et al. paper in this supplement).

In addition, there is growing evidence from community groups working on the ground in a number of countries that weaknesses in the availability of beds and personnel, combined with insufficient training in the face of growing demand, leads to a number of questionable practices: women are discharged from the labour wards too soon after delivery; practices during delivery include routine episiotomies, application of excessive fundal pressure, unnecessary oxytocin injections and other practices meant to speed up the delivery; unnecessary caesarean sections become the norm; and poorly trained personnel are unable to recognize or manage obstetric emergencies before it becomes too late to save the life of the woman (Sen and Govender 2015). This implies that policies established to improve women’s access to quality care may in some cases increase harms rather than benefits for poor women in particular (who are typically the target of these demand-side finance programmes). The evidence of disrespect and abuse during childbirth, often linked to power relationships, creates another barrier and risk for women (Department of Reproductive Health and Research, World Health Organization 2015a,b). The focus within the literature on benefits packages and gender has been on reproductive health and safe motherhood, which while important by no means does justice to the wide range of gendered needs for health services. For example, mental health needs are known to be varied across men and women, as are risks related to air pollution and suicide, to name just a few examples (WHO 2009). These are, however, rarely discussed in relation to purchasing strategies in low and middle-income countries. Equity is predominantly conceptualized in relation to income and geography.

### Governance of health financing

A recent WHO guide to conducting country-level health-financing diagnoses mentions the importance of good governance, accountability and transparency in health financing arrangements (McIntyre and Kutzin 2016). Structuring the governance of health financing institutions to ensure the engagement and perspectives of all segments of society, including different genders, is another area in which literature is lacking.

## Case study: India

Although gender analysis of health financing mechanisms and reforms is limited, it is instructive to dig more deeply into the case study of India, which has the largest population of poor women in the world (World Bank 2014a–c; Office Registrar General of India 2011). The country has high levels of gender-based inequality with a Gender Development Index of 0.795 in 2014, which places it among countries with the lowest equality in Human Development Index achievements between women and men (UNDP 2015). While India has initiated a number of health financing initiatives with the aim of increasing coverage of healthcare services to low-income groups, especially for maternal health care of women from poor households and less economically developed states of India, the gendered impact of the health financing schemes has yet to be studied in detail. The following case study draws on available evidence to make a preliminary assessment.

Very low-public investment in health, which stood at 1.05% of the GDP in 2015 (Singh and Mehta 2016), has been a feature of health care financing in India for many decades. Health care in India has been predominantly financed by household out-of-pocket expenditure for several decades, ranging from 67% in 2000 to 61% of total health expenditure in 2012 (WHO 2015b). India’s government funding for health is through taxes, and the government is also a health service provider with a network of health facilities at the primary-, secondary- and tertiary-care levels. India has a low-income and wealth tax-base and the health sector has to compete with other sectors for allocation of resources (Gudwani *et al.* 2012). Cuts to health (and other social sector) budgets are a common means of containing fiscal deficits.

Chronic funding shortfalls have resulted in a public sector characterized by shortages in service delivery points, especially in poorer states and districts; inadequate staffing; shortage of drugs; non-availability of diagnostic services; and limited range of services at the primary and secondary care levels. For example, in 2011, India had a hospital bed to population ratio of 0.7 per 1000 (World Bank 2015a), a physician to population ratio of 0.7 per 1000 (World Bank 2015b) and a nurse/midwife to population ratio of 1.7 per 1000 (World Bank 2015c), all figures well below benchmarks set by the World Health Organization.

The poor state of public sector health facilities affects women disproportionately. When services for essential health needs are not available in the subsidised government facilities, a large proportion of women are compelled to forego health care because they cannot afford to use private health care, which involves out-of-pocket expenditure.

According to India’s National Sample Survey (2004), untreated morbidity was higher among women as compared with men, especially among those in the 15–45 age group (Mukherjee and Karmakar 2008). However, the most recent round of the same survey (2014) does not indicate a gender gap (National Sample Survey Organization 2015). Smaller scale studies from the previous decade have shown steeper differences: in low-income settlements in Mumbai, untreated episodes of illness among men was 18%, and among women it was 20% without probing and 45% when they were probed (Nandraj *et al.* 2001). Among urban slum dwellers in Delhi and Chennai, 27.5% and 9% respectively of men stated financial constraints as the main reason for not seeking treatment in a 2002 study; the figures were 46% and 25% for women from the same communities (Sundar and Sharma 2002).

It is not only women from low-income households and those not engaged in paid employment who may be unable to seek health care because of financial constraints. The National Family Health Survey-3, 2005–06, reported that 40–50% of women with >12 years of education, employed for cash, and belonging to the highest wealth quintiles did not have the autonomy to decide how to spend money (IIPS, Macro International 2007).

Since the early 1990s, India has witnessed a number of policy measures that aim to create a ‘positive economic climate’ for the growth of the private health care sector. The new Bharatiya Janata Party (BJP) government that took office in 2014 has continued with the previous government’s support for the private sector in health and has introduced a few major reforms to further strengthen it (Government of India 2015). Increasing the role of the private for-private health sector has meant increasing average out-of-pocket expenditure for each health care seeking episode. Expenditure on medicines are estimated to constitute about 50–80% of treatment costs (Srinivasan 2011), and a series of changes introduced since 2010 in drug price control policies have contributed to escalating costs of essential medicines (Srinivasan *et al.* 2014).

Increasing privatization of health service provision affects women from across the socio-economic spectrum. Low-income women have to pay for all SRH services other than delivery care, while women who use private sector facilities for delivery care often incur very high out-of-pocket expenditure. A study using national survey data for 2007–08 reported the mean expenditure incurred for a normal delivery in a private health facility to be 84 USD, and for a caesarean delivery as high as 256 USD (Mohanty and Srivastava 2013).

The federal government also finances a nation-wide Social Protection Mechanism for households living ‘below the poverty line’. This is the RSBY, which is tax-funded and purchases health care from public as well as ‘empanelled’ private health care facilities. In addition, there are a number of state-government sponsored schemes in Andhra Pradesh, Himachal Pradesh, Karnataka, Kerala and Tamil Nadu, which cover secondary or tertiary health care with varying extents of financial coverage (Selvaraj and Karan 2012). India’s main social protection scheme—RSBY—covers only households living below the poverty line for a selected range of inpatient services. However, as seen from NFHS-3 data, it is not only women from poor households but also educated and employed women from middle- and upper-income households who encounter financial barriers. Failure to take this fact into account results in the deprivation of much-needed coverage of inpatient services for a section of women and men.

Studies indicate that the RSBY has increased access to care for low-income women (Cerceau 2012). However, a more gender-aware design could have removed some major barriers. For example, Rs.30 000 per annum is available for covering hospitalization for the ‘household’; however, only five members may be enrolled per household. Thus, the RSBY leaves the choice of who is to be covered to household dynamics. It has been found that girls and elderly women are more likely to be excluded when there are more than five members in a household, and overall enrolment of women is lower than that of men (Cerceau 2012). There are also non-financial barriers to utilizing the RSBY even among women who are enrolled, arising from gendered inequities. These include inadequate information on which health facilities are empanelled and what services are covered and lack of confidence to negotiate with health care providers about their entitlements under the RSBY (Cerceau 2012).

In addition, in India, there is a tax-funded Conditional Cash Transfer Scheme (CCT)—the *Janani Suraksha Yojana* (JSY)—which offers a cash incentive to women who deliver in a health facility. The eligibility criteria for receiving the cash incentive vary across states. States with a low proportion of institutional deliveries offer it to all women, while other states offer it only to women from households ‘below the poverty line’ (National Health Mission 2013). While the JSY has increased the proportion of women delivering in institutions significantly, gender-based vulnerabilities were not factored into the design of the scheme. Across all states, the scheme excludes women who already have two live births. As fertility levels are considerably higher among women from the two lowest wealth quintiles and among women with lower educational levels (IIPS and Macro International 2007), the exclusion of women with more than two live births from the JSY scheme disproportionately affects marginalized groups of women.

In states of India with a high proportion of institutional deliveries, only women from households below the poverty line, and those above 18 years of age, are eligible for the JSY. Even among those satisfying all eligibility criteria, women from the most marginalized groups tend to be excluded. For example in a Tamil Nadu study, only 25% of women who satisfied the eligibility criteria benefitted from the conditional cash transfer scheme of the state government, as caste and landowning status were significantly associated with receiving benefits. The main reasons for exclusion were difficulties encountered in producing the necessary papers to prove eligibility because of lack of information, time and contacts (Balasubramanian and Ravindran 2012).

There have also been some unexpected perverse effects from the conditional cash transfer schemes for promoting institutional deliveries. The dramatic increase in institutional deliveries in public sector health facilities has resulted in the neglect of almost all other essential SRH care, especially at primary- and secondary-care levels. Women have to seek all other SRH services from the private health sector, incurring high levels of out-of-pocket expenditure. A study of 49 women from low-income households who were hospitalized for hysterectomy in rural Tamil Nadu, for example, found that costs incurred for the surgery in private hospitals was Rs. 25 000 in a private hospital, an amount equivalent to 30 times monthly per capita expenditure in rural Tamil Nadu (approximately Rs. 850) (Balasubramanian 2011).

## Discussion

This article was based on a rapid review to examine the extent of and focal areas within the literature on health financing and gender. It is, therefore, not comprehensive but does bring out some overall findings and highlights key gaps in the health literature. Our rapid review of the literature reveals that there has been relatively little gendered analysis of health financing arrangements and, where analysis has been conducted, the focus has been on a few areas. In order to help fill this gap, Table 1 gives examples of the kinds of questions which could be approached from a gender angle within each health financing function, and a summary of the volume of work which has done in relation to these questions. Most work appears to have focused on the gender implications of user fees, and to a lesser extent on resource allocation to specific service package areas, such as mother and child health. However, other areas of potential significance, such as analysis of the gender implications of different service packages or of different provider payment mechanisms, are neglected.

**Table 1.** Gendered questions on health financing and summary of current literature

Health financing function	Examples of gendered questions	Summary of state of evidence
Revenue raising	Fairness of financial contributions: who is paying for health care? How is that changing over time How far does the burden fall disproportionately on one sex? What is the gender implication of changing revenue sources (e.g. out of pocket likely to fall heavily on women; prepaid mechanisms may be more protective)? How do different payment systems affect men and women’s access to health care? How are they affected by household arrangements (livelihoods, access to cash, decision-making power etc.) and how do they affect these in turn? What is the pattern of private and public funding and what does that mean for meeting the needs of different population groups?	This has received most attention but focussed on questions 4 and 5, especially in relation to user fees. Other areas need more probing
Risk pooling	Who is protected under different risk pooling systems (tax-based, insurance, prepaid mechanisms etc.)? How effective are the risks pools in protecting men and women against health shocks (ensuring access and also financial protection)?	This question is usually examined in relation to quintiles, but not gender
Resource allocation	How do patterns of resource allocation at different levels (national, regional, district) and within different systems and schemes affect equity of access and use for both genders, as well as quality of care? (Not just allocation of funding, but also infrastructure, human resources, etc.)	This is an important but neglected area
Purchasing	Which programmes are being prioritized for funding and how do these reflect different gender needs? Does the public/private mix serve the interests of both men and women effectively? Are gender-sensitive services being purchased (e.g. facilities which provide confidentiality, sensitivity, right staffing mix, at appropriate opening times, etc.)? Are provider payment mechanisms incentivising appropriate and high quality services for both genders?	Work has been done on resource allocation to mother and child health and sexual and reproductive health programmes but limited wider analysis (including of gender implications of different public private partnerships)
Benefits package	Is there a clear and fair entitlement to services? Are different genders equally aware of them and able to access without stigma? Do utilization patterns suggest that needs are being fairly met across the genders, or are there remaining financial and social barriers?	Not usually approached from a gender angle, but benefits packages do have gendered implications (e.g. may neglect some common male conditions, or important elements for women, such as family planning, safe abortion, infertility treatment and treatment for victims of sexual violence)
Health financing governance	Is there adequate and fair representation of different genders in health financing governance structures? Who is represented in health facility management committees, for example? Who decides on resource allocations?, etc. Does the regulatory system ensure fairness and quality of care for both genders?	This is an important but neglected area

It indicates the need for more collaboration across professional ‘silos’—not only health financing people giving more attention to gendered differences but also gender experts taking an interest in health financing arrangements and how they can help or hinder progress towards universal health coverage. Gender analysis frameworks (Morgan *et al.* 2016) could help to highlight important underlying patterns relating to access to resources, division of labour, social norms and roles, which affect health financing processes and outcomes and reproduce gender inequities at household, meso and macro levels. For example, targeted financing mechanisms reinforce women^’^s roles as vectors for child health rather than as rights-bearing citizens with comprehensive needs. This can link to other bodies of gender analysis on health systems—for example, the small but growing literature on the gendered health workforce, which puts women into lowly paid caring roles as an extension of the household division of labour (George 2008; Witter *et al.*). There is also a need to better understand specific contexts. Fragile and post-conflict states, for example, face-specific challenges, and while work is starting to emerge on how to develop more gender-sensitive health financing policies (Ssali *et al.* 2016), more work is needed. Given the political nature of UHC decisions, the political economy as well as technical research should be prioritized. The effects and interactions of technical interventions in complex systems are inherently unpredictable, but clear policy goals and an openness to monitor and respond to unintended consequences are important starting points.

In reviewing the performance of different health fund-raising and pooling mechanisms, it is evident that some are much better than others both in terms of improving efficiency and also in meeting the equity requirements, including reducing gender inequalities, implicit in the UHC definition. Specifically it is evident that compulsory public financing mechanisms (in countries such as Thailand, Brazil or Sri Lanka) outperform private voluntary mechanisms because only the former can facilitate the cross-subsidies necessary to cover the poor and vulnerable (Kutzin 2012; Rottingen et al 2014).

However, one should not assume that building a health financing system based on compulsory public financing mechanisms will be sufficient to reduce gender inequities. This is because health-financing reforms are inherently political processes and there will always be a tendency for powerful groups to capture a disproportionate proportion of benefits and minimize costs for themselves. This can be seen in the tendency for governments to establish health insurance schemes that cover people working in the formal sector—which disproportionately benefits civil servants and men. Linking health coverage to employment status originated in Europe in the 19th and early 20th centuries when health benefits were prioritized for men because states wanted a healthy male workforce to fight wars and work in rapidly industrializing economies. This is incompatible with the more modern approaches which emphasize women’s contribution to the workforce, as well as a universal right to health, as illustrated by the UN Declaration on Human Rights, which recognizes the wider social and economic benefits of women’s health (Langer *et al.* 2015).

Due to large political pressure from civil society organizations and excluded populations, many countries have moved away from this selective approach, to establish coverage systems built on a universal entitlement to services and financial protection (Evans *et al*. 2012; Rannan-Eliya and Sikurajapathy 2009). As countries move towards UHC they should ensure that everybody is covered in an equitable manner and that vulnerable groups, notably women, are prioritized from the outset. This preferred route to UHC has been called ‘progressive universalism’ by the Lancet Commission on Investing in Health, which called for a grand convergence of health indicators (and therefore an elimination of inequalities) by 2035 (Jamison *et al.* 2013).

## Conclusion

Our review has drawn attention to two main gaps: research gaps (areas where collaboration across disciplines could yield more gender-responsive analysis) but also the more substantive gaps in health coverage, and the contribution which health financing can make to closing them. The pattern of health financing in countries like India has clear gendered implications, with women at a relative disadvantage. A more gender-equitable approach to health financing would include, for example: tax-based public financing of health care services with resources mobilized from progressive taxation of income and wealth; firm action by governments to regulate the private sector in health, especially in the area of price controls; attention to coverage of all sections of women when implementing health financing reforms (e.g. social insurance, micro-insurance); and social protection schemes that go beyond women from households below the poverty line and with packages covering services across the life-cycle (not just reproductive health).

Unless explicit attention is paid to gender and its intersectionality with other social stratifiers, through explicit protection and careful linking of benefits to needs of target populations (e.g. poor women, unemployed men, female-headed households), movement towards UHC can fail to achieve gender balance or improve equity, and may even exacerbate gender inequity. Political trade-offs are made on the road to UHC and the experiences and interests of less powerful groups, which can include women and children, are not necessarily given priority. Countries should adopt an equitable approach towards achieving UHC and through progressive universalism prioritize high need groups and those requiring additional financial protection, in particular women and children.

## Footnotes

1 Household where at least one, but not all members are covered by private health insurance (Govender *et al.* 2014).
